# Low Cost Fabrication of Si NWs/CuI Heterostructures

**DOI:** 10.3390/nano8080569

**Published:** 2018-07-25

**Authors:** Maria José Lo Faro, Antonio Alessio Leonardi, Dario Morganti, Barbara Fazio, Ciro Vasi, Paolo Musumeci, Francesco Priolo, Alessia Irrera

**Affiliations:** 1 MATIS IMM-CNR, Institute for Microelectronics and Microsystems, Via Santa Sofia 64, 95123 Catania, Italy; mariajose.lofaro@ct.infn.it (M.J.L.F.); antonio.leonardi@ct.infn.it (A.A.L.); francesco.priolo@ct.infn.it (F.P.); 2 IPCF-CNR, Institute for Chemical and Physical Processes, Viale F. Stagno D’Alcontres 37, 98158 Messina, Italy; dario.morg@hotmail.it (D.M.); fazio@me.cnr.it (B.F.); vasi@ipcf.cnr.it (C.V.); 3 Department of Physics and Astronomy, University of Catania, Via Santa Sofia 64, 95123 Catania, Italy; paolo.musumeci@ct.infn.it; 4 INFN Section of Catania, Via Santa Sofia 64, 95123 Catania, Italy; 5 Superior School of Catania, Via Valdisavoia 9, 95123 Catania, Italy

**Keywords:** silicon nanowires, heterostructures, CuI, silicon

## Abstract

In this paper, we present the realization by a low cost approach compatible with silicon technology of new nanostructures, characterized by the presence of different materials, such as copper iodide (CuI) and silicon nanowires (Si NWs). Silicon is the principal material of the microelectronics field for its low cost, easy manufacturing and market stability. In particular, Si NWs emerged in the literature as the key materials for modern nanodevices. Copper iodide is a direct wide bandgap *p*-type semiconductor used for several applications as a transparent hole conducting layers for dye-sensitized solar cells, light emitting diodes and for environmental purification. We demonstrated the preparation of a solid system in which Si NWs are embedded in CuI material and the structural, electrical and optical characterization is presented. These new combined Si NWs/CuI systems have strong potentiality to obtain new nanostructures characterized by different doping, that is strategic for the possibility to realize *p-n* junction device. Moreover, the combination of these different materials opens the route to obtain multifunction devices characterized by promising absorption, light emission, and electrical conduction.

## 1. Introduction

Semiconducting nanowires arise as innovative materials in many different fields due to their improved electrical [1,2], optical [3,4], and mechanical [5,6] properties. Among other semiconductors, silicon is of crucial interest for industrial applications for its low cost, easy manufacturing, and stability, prompting the realization of Si nanowires as promising resources to be employed in modern nanodevices [7,8,9,10,11,12,13].

Although several growth techniques have been reported [14,15,16,17,18,19,20,21], silicon nanowires (Si NWs) fabrication is still quite complex and challenging. The vapour-liquid-solid (VLS) approach is the most commonly adopted technique for the synthesis of Si NWs through the aid of a metal (or metal alloy) seed catalyst that induce the wires formation upon Si precipitation when above the Me/Si alloy eutectic temperature [14]. Even though widely diffused, VLS presents several drawbacks, mainly (i) high process temperatures, (ii) formation of NWs bundles, (iii) poor control on the structural features and (iv) non-uniform doping which limit the real integration of VLS NWs in new devices. Moreover, the choice of the metal catalyst is crucial in order to determine the NWs properties and a large number of attempts have been reported in order to circumvent the detrimental effect of catalyst impurities incorporation into device [19,20]. Other top-down approaches such as lithography and reactive ion etching are also available, allowing the controlled synthesis of Si NWs with defined structural parameters [15,16,17,18]. However, these methodologies require the iteration of several processes, resulting extremely expensive in both terms of cost, technologies and time. In addition, RIE damage the Si NWs surface causing recombination losses detrimentally affecting the performances for all industrial applications.

A valid alternative for the synthesis of Si NWs is given by the metal assisted chemical etching (MACE) based on the selective oxidation and etching of Si wafers occurring at the metal/Si interface [22,23,24]. The major advantage of MACE synthesis is the realization of high density arrays of vertically aligned Si NWs with high aspect ratio [25,26]. Another crucial aspect of MACE is its fine control over the structural properties of Si NWs: the NWs length can be tuned with great accuracy simply varying the etching time while the radius and density are interrelated parameters defined by the metal precursor concentration. Unlike VLS, the whole chemical process is performed at room temperature, hence, the metal catalysts are not incorporated into the wires. Si NWs produced via the top-down approach inherit the same doping of the starting bulk material. This approach is maskless, low-cost, and implementable with complementary metal-oxide semiconductor (CMOS) technology, allowing the NWs fabrication on a large scale with controlled structural properties. Indeed, due to their improved properties, Si NWs realized by MACE have been used for a different range of applications [27,28,29,30,31,32].

A crucial aspect for the realization of multifunctional devices is to combine the properties of different materials, such as absorption, light emission and electrical conduction, in order to improve the overall response of a device [30,31,33,34,35,36,37,38,39]. Due to their very large aspect ratio and their physical properties, Si NWs realized by MACE stand up as a promising platform for novel heterostructures with great impact for optoelectronics and photovoltaics. Several transparent conducting layers with *n*-type conduction are already available, such as aluminum zinc oxide (AZO) and Indium tin oxide (ITO). However, only a few materials meet both the requisite of *p*-conductivity and transparency and a great deal of effort has been employed in this research field. The main advantage of copper iodide (CuI) is related to its synthesis at low temperature without the need of any seed layer, which guarantee an easier integration with 1D nanostructure for device manufacturing. As a complimentary material to Si NWs, we investigated the properties of CuI [40]. Copper iodide is a direct wide bandgap *p*-type semiconductor employed for several applications as a transparent hole conducting layers for dye-sensitized solar cells [41,42,43], light emitting diodes [44,45], and for environmental purification [46].

Moreover, the CuI has the great advantages that can be infiltrates in liquid form and then precipitates in solid phase at a temperature of 80 °C, compatible with the microelectronic industry. 

In this work, we demonstrate the realization of a novel material based on the decoration of silver salts MACE Si NWs with CuI deposited by using chemical bath deposition assisted with airbrush spray coating, a low cost approach compatible with the standard Si technology.

We investigated the deposition conditions leading to the formation of Si NWs/CuI heterostructures (HS) in two different configuration: core-shell HS and Si NWs embedded in a CuI matrix with a top capping layer. Therefore, the possibility to realize *n*-type doped Si NWs completely covered by the CuI, which is a *p*-type semiconductor, open the route toward the realization of innovative *p-n* junction strategic for future nanodevices.

## 2. Materials and Methods

### 2.1. Materials and Chemicals

Single crystal silicon wafers (100) with *n*-type doping (resistivity ~1–5 Ω·cm) and 500 μm of thickness were acquired by Siegert Wafer (Siegert Wafer GmbH, Aachen, Germany). Hydrofluoridric acid (HF) 50% and acetonitrile (CH_3_CN) were purchased by Sigma Aldrich (Sigma Aldrich SRL, Milano, Italy), while copper iodide (CuI) powder, silver nitrate (AgNO_3_), and nitric acid (HNO_3_) were acquired by Scharlau (Scharlab Italia srl, Milano, Italia). All of the aqueous solutions were prepared by Milli-Q deionized water (resistivity ~18 MΩ·cm).

### 2.2. Silicon NWs Growth

**Figure 1:**
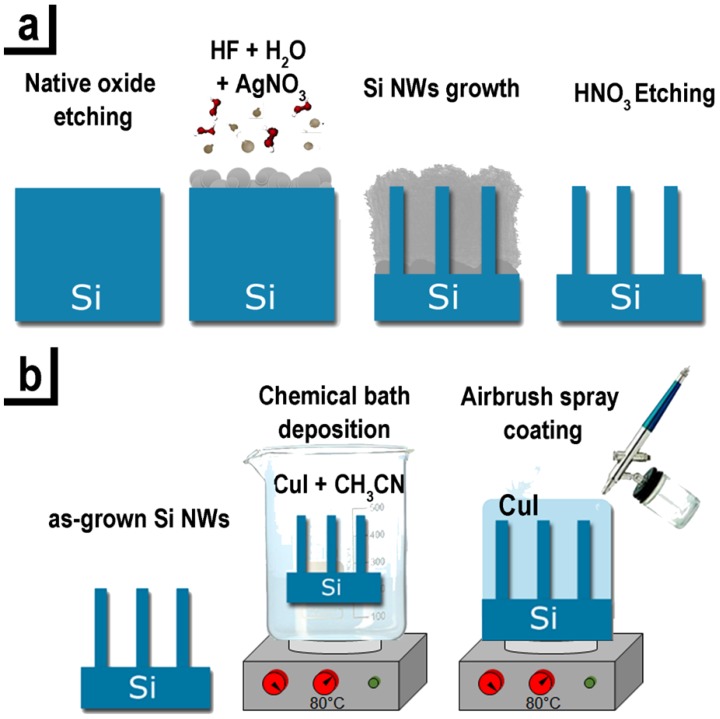
Schematic of Si NWs/CuI heterostructures realization: (**a**) Synthesis of Si NWs by AgNO_3_ metal assisted chemical etching; (**b**) Deposition of CuI by chemical bath and airbrush spray coating.

The Si NWs were realized by the silver salts MACE approach [47] schematized in Figure 1a:

- The Si wafer surface was initially cleaned from the presence of organic contamination with an ultraviolet (UV) ozone treatment. Subsequently, in order to remove the silicon oxide, the samples were immersed in a solution of 5% HF and 95% of H_2_O.
- The oxide-free Si substrates were immersed in an aqueous solution of 40% AgNO_3_ and HF 20% and the dissolved Ag salts form a precipitation of small silver nanoparticles (NPs) onto the Si substrate.
- During the process the Ag NPs act as a catalyst leading to the oxidation of the underneath silicon that is etched by the hydrofluoric acid, leading to the formation of Si NW for the unetched Si regions.
- The network of silver dendrites formed during the growth was removed by a 10 min chemical bath of HNO_3_.

Each step of the process is performed at room temperature preventing the diffusion of the metal catalyst into the nanostructures. The method is based onto the dissociation of the silver salts, realizing a precipitation of Ag nanoparticles that are randomly distributed on the silicon surface. Under the Ag NPs covered region there is the Si oxidation and it is selectively etched by the HF in the solution. By changing the concentration of the AgNO_3_ that determines the density and size of the precipitate Ag nanoparticles onto the Si substrate is possible to tune the structural characteristics of the NWs array (density, spacing, and filling factor).

### 2.3. Realization of Si NWs/CuI Heterostructures

A crucial point for the realization of Si NWs CuI heterostructures is the uniformity of the surface coverage. In fact, due to their very high aspect ratio and their micrometer vertical profile is quite complex to fully decorate these type of nanostructures with a good uniformity from the top to the bottom. In order to obtain this strategic point, we optimized the following procedure.

- The Si NWs samples were cut in 3 × 3 cm^2^ pieces to guarantee the uniformity of the CuI coverage.
- Chemical bath deposition (CBD). In order to obtain the core shell Si NWs/CuI HS the as-grown Si NWs arrays were immersed in a solution of 63 mM of CuI powder dissolved in acetonitrile heated at 80 °C for 20 min hold by a clip. The solution was stirred at 70 rpm with a magnetic stirrer. The CuI solution diffuses inside the interstices between the Si NWs and the HS are realized by the CuI precipitation in its solid phase. Finally, the samples were dried with a nitrogen flux.
- Airbrush spray coating (ASC). Subsequently the CBD, the sample was put on a hot plate at 80 °C covered with an aluminum foil where was subjected to an airbrush spray coating with the same CuI solution, previously heated at 80 °C.

In Figure 1b all the realization steps of the the Si NWs/CuI HS described in these points are depicted. 

**Figure 2:**
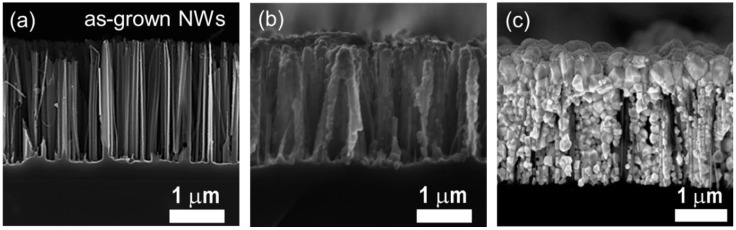
Cross-section SEM images displaying (**a**) a 2-µm long as-grown Si NWs obtained by AgNO_3_ MACE, (**b**) a core-shell Si NWs/CuI heterostructure realized by chemical bath deposition for 20 min in a 63 mM solution of CuI diluted in acetonitrile, and (**c**) the Si NWs/CuI heterostructure obtained after both chemical bath deposition and airbrush spray coating.

Core-shell Si NWs/CuI HS are formed by CBD (Figure 2b), while the realization of embedded system is engineered by combining CBD and ASC procedure, guarantying the formation of a CuI matrix surrounding the NWs with a top coating layer whose thickness dimension is determined by the volume amount of the sprayed solution (Figure 2c). 

### 2.4. Structural and Optical Characterization Methods

Structural and chemical characterization of the Si NWs/CuI HS samples were performed by a Zeiss Supra 25 field-emission scanning electron microscope equipped with an energy-dispersive X-ray (EDAX) detectors (EDAX, Mahwah, NJ, USA) for EDX analysis. SEM characterization were analysed by software analysis using Gatan Digital Microscopy suite (Gatan, Pleasanton, CA, USA). The stoichiometric composition was measured by the Rutherford backscattering spectrometry (RBS, High Voltage Engineering Europa B, Amersfoort, The Netherlands). A He^+^ beam was generated by a HVEE Singletron accelerator system (High Voltage Engineering Europa B, Amersfoort, The Netherlands) at an energy of 2 MeV and collided onto the samples with a circular beam spot of 1 mm in diameter. The loss of He^+^ energy was investigated by a multichannel analyser in a back scattering configuration acquiring the scattered He^+^ ions at an angle of 165° with respect to the ion beam direction. The RBS spectra simulations were obtained through SimNRA software (Max-Planck-Institut für Plasmaphysik, Garching, Germany). The X-ray diffraction measurements were performed through a Bruker AXS D5005 diffractometer (Bruker, Billerica, MA, United States) using the Cu-K_α_ radiation in a θ/2θ configuration. The analysis were performed with an incident angle of 5° and in grazing incidence configuration with an angle of 0.5° and the crystalline phase identification was obtained by the database Bruker DIFFRAC.SUITE (reference γ-CuI DB0018105, Bruker, Billerica, MA, USA). 

Reflectance measurements were carried out by using an ultraviolet-visible (UV–VIS) Lambda2 Perkin-Elmer double-beam spectrometer (Perkin-Elmer, Waltham, MA, USA) equipped with a specular reflectance accessory. Measurements were performed in the wavelength range 200–1100 nm. Current Intensity versus voltage (I-V) measurements were carried out with a two-point setup using a Keithley 4200 semiconductor characterization system (Keithley, Cleveland, OH, USA) at room temperature. The photogeneration measurements were realized under illumination with a 150 W halogen lamp. The photoluminescence (PL) measurements were acquired through a micro-spectrometer (HR800 Horiba-Jobin Yvon, Kyoto, Japan) in back-scattering configuration equipped with a CCD. In particular, the sample was excited by using the 364 nm line of an Ar^+^ laser at a power of about 90 µW onto the sample. The excitation light was focused and then collected by the sample through a fluorinated 60× UV Olympus objective (NA = 0.9).

## 3. Results

### 3.1. Structural Characterization of Si NWs/CuI Heterostructures

Figure 2a shows a dense forest (about 10^10^ NWs/cm^2^) vertically aligned 2 µm long Si NWs array synthesized on a (100)-oriented *n*-type Si substrate by silver salts MACE with etching time of about 10 min. These Si NWs have diameter ranging from 30 to 80 nm, a spacing ranging from 30 nm to 700 nm and a filling factor of about 30% as investigated by software analysis of the SEM images. Si NWs arrays with length of 2 μm and *n*-type doping were used for the realization of the heterostructures upon the selective removal of Ag NPs residues and are from now on referred as “as-grown” Si NWs. 

The CuI decoration conditions were optimized in order to vary the morphology of the Si NWs/CuI heterostructures. We demonstrate the realization of two different morphologies: (i) a radial core-shell system of Si NWs wrapped by CuI nanoparticles (Figure 2b) and (ii) a fully embedded system were Si NWs are surrounded into a CuI matrix covered with a uniform top layer (Figure 2c). 

The cross section SEM microscopies of as grown Si NWs, core-shell Si NWs/CuI system and of a heterostructure of Si NWs embedded into the CuI matrix are compared in Figure 2. It is worth noticing that the Si NWs array is not damaged by the decoration processes. Moreover, both HS configurations show that we are able to fully decorate Si NWs array along the whole vertical profile, from their tips to the bottom with different HS morphologies.

Si NWs/CuI core-shell system shown in Figure 2b is realized by chemical bath deposition at 80 °C for 20 min (without spray coating) and the presence of small CuI NPs with average dimension of 100 ± 30 nm around the NWs surface from the top to the bottom is clearly attested. Indeed, the CuI salts dissolved in the acetonitrile solution precipitate onto the Si NWs at a temperature of about 80 °C realizing a uniform coverage of the NWs length. In order to obtain the Si NWs/CuI embedded HS shown in Figure 2c, a two steps procedure was optimized: a CBD is performed at 80 °C for 20 min under stirring, followed by airbrush spray coating of the heated sample with 6 mL of CuI solution (see experimental Section 2.3). This two-steps procedure consent to decorate Si NWs with CuI NPs fully covering the NWs surface by CBD, while the sprayed solution allows the formation of CuI crystallites with a diameter of 150 nm surrounding the NWs. Moreover, a continuous CuI capping layer with a diameter of CuI NPs from 300 nm to 600 nm can be deposited onto the Si NWs tips by spraying the solution, which is of interest to electrically address the system. According to the applications, the thickness of the capping layer can be varied by controlling the volume of the sprayed solution. We optimized the uniform decoration of Si NWs arrays with fixed length of 2 µm, realizing the Si NWs/CuI embedded heterostructure shown in Figure 2c by using 6 mL of sprayed solution. The CuI capping film has thickness of about 600 ± 100 nm with an increased grain size ranging from 300 nm up to 600 nm.

By using this low-cost and fast approach, we demonstrated the realization of Si NWs/CuI HS with different morphologies through a method compatible with the Si technology. 

A crucial aspect for the realization of NWs heterostructures is the demonstration of a full and uniform coverage system, which is of strategic importance to improve the device performances. 

**Figure 3:**
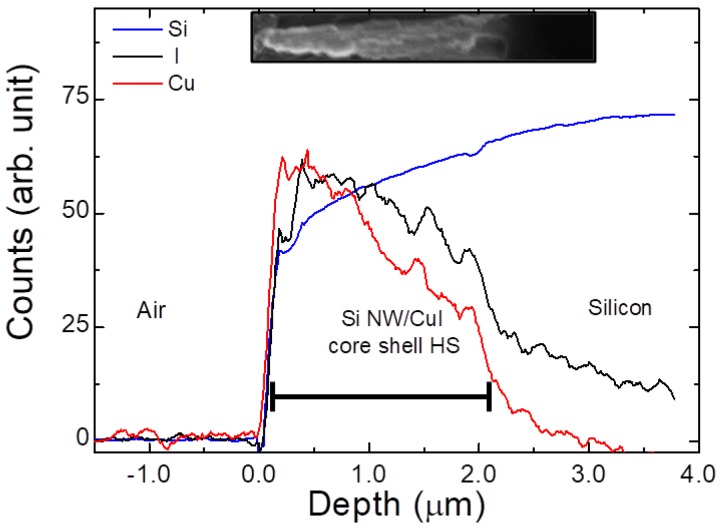
EDX analysis on the cross-section profile of the core-shell Si NWs/CuI heterostructure reporting the Si-Kα, Cu-Lα and I-Lα X-ray emissions displayed in blue, red and black lines, respectively.

As elicited, the critical step for the formation of NWs HS is first step of the chemical bath deposition. Therefore, the presence of CuI along the NWs vertical profile was investigated by EDX spectroscopy. The EDX analysis is realized on the sample obtained only with the chemical bath deposition (Figure 2b) without the CuI capping layer. Figure 3 shows the EDX profilometry along the core-shell system (SEM shown in the inset) reporting the X-ray emission lines of Si-Kα (1.74 keV), Cu-Lα (0.93 keV) and I-Lα (3.9 keV) displayed in blue, red and black, respectively. Although the Si NWs are fully covered, the EDX analyses demonstrate that the CuI concentration at the bottom of the NWs is decreased by 42%, as expected from the decoration of high-aspect ratio nanostructures. Conversely, Si-Kα signal (blue line) has a minimum onto the NWs tips and increases moving towards the bulk with the opposite trend observed for the CuI. Although EDX analysis provides important qualitative information, this technique has a lack of spatial resolution due to the huge pear of interaction extended inside the material. Indeed, at about 3 µm is still visible the signal of the CuI coming from the pear of the overlying regions covered with CuI. The Si NWs have length of 2 µm and the EDX performed along their vertical profile demonstrate the full coverage of their vertical profile with CuI in agreement with SEM characterization.

**Figure 4:**
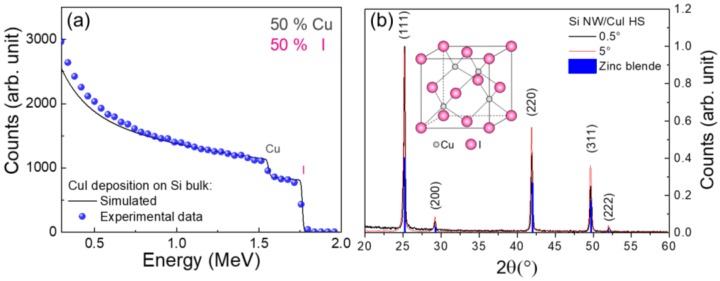
(**a**) Rutherford backscattering spectrometry of a CuI layer deposited onto a Si bulk substrate with CBD and spray coating. Experimental data (blue dots) were fitted with a simulated layer with composition Cu_0.5_I_0.5_ obtained from SimNRA (black line). (**b**) XRD spectra for the Si NWs/CuI heterostructure obtained at 0.5° (black line) and 5° (red line) compared to the diffraction peaks associated to the CuI zinc blend structure reported from database (blue lines). The inset shows a scheme of γ-CuI zinc blende structure.

Rutherford backscattering characterization was performed in order to confirm the stoichiometry of the deposited CuI. RBS provides a quantitative elemental composition of thin films through ion beam analysis. However, the energy loss of the backscattered ions are heavily affected by the presence of surface roughness, voids, and precipitates, altering the shape of the spectral signature of chemical elements. In order to avoid such problematics, RBS measurements were performed on a CuI layer deposited onto Si bulk substrate with the same approach adopted for the NWs decoration described in the experimental Section 2.3. The experimental RBS spectrum of CuI deposited onto Si bulk is reported as blue dots in Figure 4a. The black line corresponds to the simulated spectrum obtained from a superficial layer with composition of Cu_0.5_I_0.5_. From the comparison it can be observed the two RBS spectra have the same composition attesting a stoichiometry 50% of Cu and 50% of I, in agreement with what is expected. However, a slight mismatch is attested for both elements at the backscattering energy edges ascribed to the presence of a certain degree of superficial roughness due to the formation of CuI crystalline domains onto the Si bulk, as well as on Si NWs. 

The stoichiometry obtained from this RBS conducted on CuI deposited onto Si bulk can generalized to the case of CuI deposited on Si NWs since the same experimental conditions were used.

Apart from the stoichiometry, the physical properties of a material are determined by its crystalline phase. CuI is a binary inorganic compound presenting a rich crystal phase diagram ranging from zinc blende (γ-CuI) below 390 °C, wurtize (β-CuI) between 390–440 °C, and (α-CuI) with a halite structure above 440 °C, each one characterized by a different energy gap [48]. We investigated the crystal properties of Si NWs/CuI HS by XRD characterization, as shown in Figure 4b. 

The Si NWs/CuI embedded HS XRD spectra were acquired at incident angle of 5° (red line) and 0.5° (black line) in order to test the crystalline composition of the material deposited deep inside the NWs interstices and onto their surface, respectively. Both XRD spectra show the same diffraction peaks associated to (111), (200), (220), (311), and (222) crystalline orientations characteristic of the γ-CuI zinc blende structure [49], whose scheme is depicted in the inset. The position and relative intensities of these diffraction peaks are the same of those achieved from the database (γ-CuI DB0018105). Therefore, the CuI present in the Si NWs/CuI heterostructure is in stable zinc blende form, as expected from the low temperature adopted during the deposition. This result is a strong confirmation of the efficiency of the decoration procedure, attesting that CuI is present with the same γ-phase deep inside the dense NWs forest as well as on their tips. Both Si NWs/CuI HS morphologies are in the same γ-phase since the identical temperature of 80 °C was used during the two growth processes. Moreover, impurities diffraction peaks are not present in the XRD patterns, confirming the high purity and quality of the synthesized products, strategic for future industrial applications.

### 3.2. Si NWs/CuI Embedded Heterojunction for Photovoltaics

The γ-CuI has a characteristic wide and direct energy bandgap of about 3.1 eV at room temperature, showing good transparency overall the visible region [50]. For these reasons, γ-CuI is of strategic interest for application in photovoltaic (PV). Moreover, vertical array of Si NWs recently emerged as innovative template for PV due to their improved light trapping. Indeed, several groups demonstrated the low reflectance behavior of solar cells textured with ordered [10,17] and disordered [4,51] Si NWs arrays.

In this scenario, the low cost and Si industrially-compatible fabrication of Si NWs/CuI heterostructures arise as highly appealing for PV applications. The PV characterization are performed on a *n-p* heterojunction of Si NWs/CuI. Si NWs are embedded into CuI matrix with a CuI continuous layer with thickness of about 600 nm on the top. This CuI capping layer improve the stability of the front electrical contact (as visible in Figure 2c). Probably the presence of the CuI capping layer may limit the PV performances due to the partial carriers’ recombination inside this layer. However, the PV measurements without capping layer are not very stable making necessary the realization of this CuI layer.

**Figure 5:**
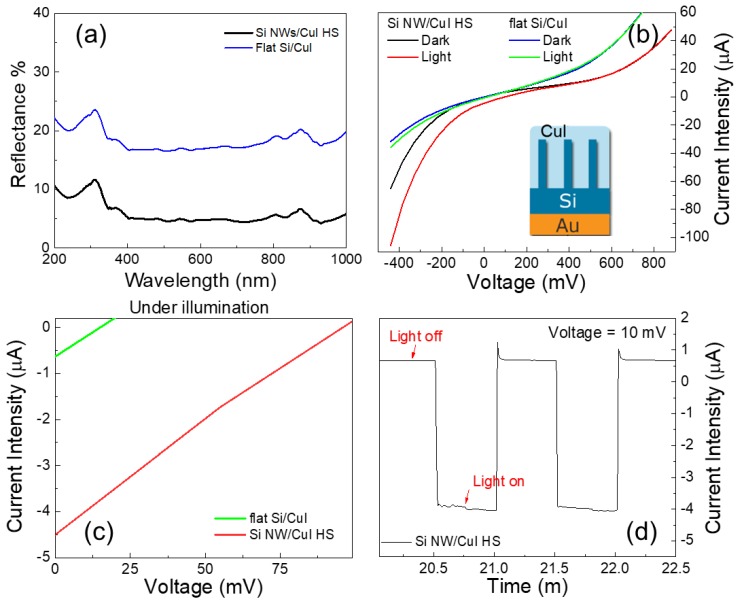
(**a**) Reflectance measurements of flat Si /CuI and Si NWs/CuI junctions are reported in blue and black, respectively (**b**) I-V characteristic of both Si/CuI and Si NWs/CuI *n-p* heterojunction investigated under dark and light illumination conditions. The inset shows the schematic of the Si NWs/CuI device contacting during the I-V characterization. (**c**) PV output characteristic of the *n-p* Si/CuI and Si NWs/CuI devices tested under light illumination are show in green and red, respectively. (**d**) Current intensity measured in the device biased at 10 mV under dark/light pulses modulated by intervals of 25 s.

In order to demonstrate the advantaged of Si nanowire with respect to a flat Si bulk substrate we report the reflectance of both system as shown in Figure 5a. The reflectance of flat Si/CuI is shown in blue. A pronounced peak is observed at about 270 nm ascribed to the reflective behaviour typical of Si in this range. The same peak is also visible for Si NWs/CuI spectrum showed in black. The light trapping efficiency is increased for Si nanowires due to the surface texturization causing the suppression of the reflectance below value of 10% across the UV–VIS region due to the multiple scattering within the NWs array.

The PV devices were electrically addressed with a direct contact onto the CuI transparent and conductive capping layer, while the back contact was taken from the Au metal layer deposited onto the back of Si (see inset in Figure 5b). The I-V characteristics of Si NWs/CuI and the flat Si/CuI junctions under dark and light illumination with halogen lamp are compared in Figure 5b for the extended voltage region. The inset shows a schematic of the I-V measurements performed on the Si NWs/CuI device. The I-V curves under light illumination are shifted to negative current value demonstrating the collection of photogenerated electron-hole pairs in both devices. It is worth noticing that the Si NW/CuI trend under both dark and light illumination is slightly different from the PV cell ideal curve better resembled from the flat Si/CuI device. This effect is due the combined effects of internal and external resistances. However, it can be clearly attested from the comparison of the curves that the Si NWs/CuI junction is more efficient in terms of photogenerated current. 

In Figure 5c the I-V segment corresponding to the photogenerated current is shown by the green and red lines for flat Si/CuI and Si NWs/CuI devices, respectively. It is clearly observable that the area of photogenerated current is higher for the NW by a factor of 25 with respect to the Si bulk due to the efficient light trapping in the Si NWs/CuI junction, highlighting the advantage use of Si NWs arrays. Short-circuit current (I_sc_) of about −4.5 µA, open circuit bias (V_oc_) of 96 mV and a fill factor (FF) of 0.25 were measured for the Si NWs/CuI device the I-V trend under illumination. The photocurrent trends show linear behaviours which are typical of PV cells with high series resistance (R_s_) and low shunt resistance (R_sh_).

As well reported from literature, series resistance in a solar cell is strongly affected the current motion between the emitter and base regions (CuI/Si NWs) and by the contact resistances. High R_s_ values are responsible for a marked reduction of the fill factor and the short-circuit current, as in this case. Additionally, the low shunt resistance due to manufacturing defects may cause significant power losses by leakage current introduced by the presence of recombination centres. Indeed, a high total resistance of about 117 kΩ was measured contacting the device with a digital multimeter in the dark configuration, confirming the detrimental effects of both series and shunt resistances. We tested the stability of the photogenerated current for the Si NWs/CuI heterojunction biased at 10 mV by an alternating cycle of dark and light pulses with durations of 25 s for a prolonged period of 30 min. As an example, in Figure 5c are displayed two light on/off cycles where a stable photocurrent of −4 µA is reported. 

### 3.3. Photoluminescence of Si NWs/CuI Embedded Heterojunction

The photoluminescence (PL) measurements of the Si NWs/CuI embedded heterojunction was carried out in order to investigate the presence of defects affecting the I-V performance of the device.

**Figure 6:**
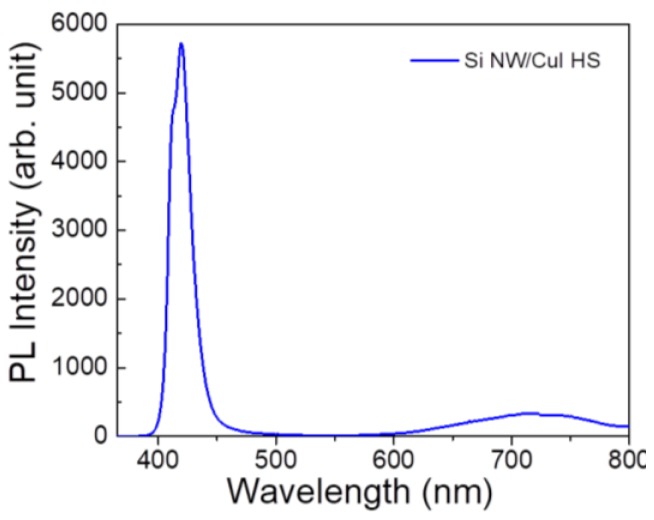
Room temperature photoluminescence spectrum of Si NWs/CuI embedded heterostructure acquired at the excitation wavelength of 364 nm.

The PL spectrum shown in Figure 6 is characterized by an intense emission in the blue region and a weak broad red emission band. The bright blue emission can be ascribed to the sum of two PL peaks: one at about 411 nm associated to the direct recombination of free excitons and the 419 nm one due to Cu vacancies [50,52]. The red emission due to I vacancies is centred at about 719 nm, in perfect agreement with the literature [53]. All three PL contributions are characteristic of CuI emission and no emission from our Si NWs is attested, as expected because the diameter of Si NWs is not compatible with quantum confinement effects. Therefore, it is possible to tune the intensity of the defects PL peaks by changing the thickness of the CuI capping layer. In fact, by changing the thickness of the capping layer is possible to vary the density of the emitter defects. 

The presence of Cu vacancies in the CuI lattice is responsible for the *p*-type conductivity of such materials, which is strategic for the realization of a transparent acceptor layer. Theoretical works from Wang et al. suggest that I vacancies introduce deep recombination centres into the energy gap [46]. The presence of I vacancies is clearly attested from the red emission band in PL spectrum and may be responsible for the low shunt resistance detrimentally affecting the I-V characteristics. Conversely, the bright CuI emission in two different range is great interest for the realization of low-cost light emitting sources and the CuI deposition onto our Si NWs make this system implementable with the Si technology [45]. 

## 4. Conclusions

In this paper, the realization of Si NWs/CuI heterostructure is demonstrated by a cheap approach compatible with the microelectronic industry. Si NWs/CuI core-shell and embedded systems were fabricated, demonstrating the capability to vary the morphologies of the heterostructures according to the applications. The structural characterizations attest the uniform coverage of Si NWs with CuI crystallites in the zinc blende phase, free from other element impurities. 

The realization of a *n-p* heterojunction obtained combined Si NWs/CuI systems is demonstrated and its photovoltaic performances were analyzed and correlated to the defects presence investigated by photoluminescence. The possibility to engineer the defects present in Si NWs/CuI heterostructures could pave the way for the fabrication of innovative multifunction Si-based devices for photonics and photovoltaics applications.
